# Yuzurimine from of *Daphniphyllum macropodum* Miq.

**DOI:** 10.1107/S1600536810041188

**Published:** 2010-10-31

**Authors:** Ying Cheng, Xing-Jin He

**Affiliations:** a College of Science, Sichuan University, People’s Republic of China; b Leshan Teachers College, People’s Republic of China

## Abstract

The title compound, C_27_H_37_NO_7_, is a *Daphniphyllum* alkaloid isolated from a branch of *Daphniphyllum macropodum* Miq. All of the five-membered rings adopt envelope conformations while the six- and seven-membered ring adopt chair conformations. Classical inter­molecular O—H⋯O and weak C—H⋯N hydrogen bonds are present in the crystal structure.

## Related literature

For the chemical structure of the title compound established from NMR and MS data, see: Li *et al.* (2009 ▶). For structures of *Daphniphyllum* alkaloids, see: Yamamura & Terada (1976 ▶); Kubota *et al.* (2006 ▶).
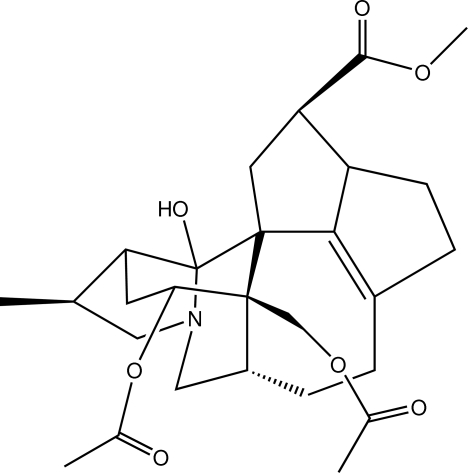


## Experimental

### 

#### Crystal data

C_27_H_37_NO_7_
*M* *_r_* = 487.58
Orthorhombic, 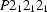

*a* = 9.5980 (3) Å
*b* = 9.7437 (2) Å
*c* = 26.0986 (6) Å
*V* = 2440.74 (11) Å^3^
*Z* = 4
Mo *K*α radiation
μ = 0.10 mm^−1^
*T* = 294 K
0.60 × 0.45 × 0.20 mm

#### Data collection

Oxford Xcalibur diffractometer with an Eos CCD detector
8335 measured reflections
2836 independent reflections
2236 reflections with *I* > 2σ(*I*)
*R*_int_ = 0.019

#### Refinement

*R*[*F* ^2^ > 2σ(*F* ^2^)] = 0.044
*wR*(*F* ^2^) = 0.120
*S* = 1.04
2836 reflections
317 parameters
H-atom parameters constrained
Δρ_max_ = 0.48 e Å^−3^
Δρ_min_ = −0.19 e Å^−3^

### 

Data collection: *CrysAlis PRO CCD* (Oxford Diffraction, 2009 ▶); cell refinement: *CrysAlis PRO CCD*; data reduction: *CrysAlis PRO RED* (Oxford Diffraction, 2009 ▶); program(s) used to solve structure: *SHELXTL* (Sheldrick, 2008 ▶); program(s) used to refine structure: *SHELXTL*; molecular graphics: *SHELXTL*; software used to prepare material for publication: *SHELXTL*.

## Supplementary Material

Crystal structure: contains datablocks I, global. DOI: 10.1107/S1600536810041188/xu5044sup1.cif

Structure factors: contains datablocks I. DOI: 10.1107/S1600536810041188/xu5044Isup2.hkl

Additional supplementary materials:  crystallographic information; 3D view; checkCIF report

## Figures and Tables

**Table 1:** Hydrogen-bond geometry (Å, °)

*D*—H⋯*A*	*D*—H	H⋯*A*	*D*⋯*A*	*D*—H⋯*A*
O1—H1⋯O3^i^	0.82	2.43	3.216 (3)	161
C22—H22*B*⋯N1^ii^	0.96	2.41	3.354 (4)	169

Symmetry codes: (i) 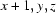; (ii) 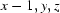.

**Fig. 1.**
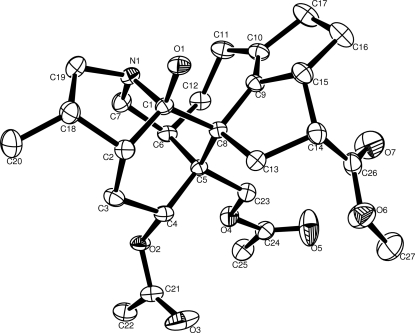
The molecular structure of the title compound with 30% probabiliy displacement ellipsoids for no-H atoms. H atoms have been omitted clarity. Dashed line indicates hydrogen bonding.

## supplementary crystallographic information

### Comment

The title compound, yuzurimine, was previously isolated from *Daphniphyllum macropodum* Miq. (Li *et al.* 2009), and its structure was established from the NMR and MS data. In our recent investigation, it was isolation from the branch of *Daphniphyllum macropodum* Miq. collected in the Emei Mountain, Sichuan Province of China in 2008. Its crystal structure is reported here.

The molecular structure of the title compound is shown in Fig. 1. Six-membered ring A (C1/C2/C3/C4/C5/C8) adopts chair conformation; Six-membered heterocyclic ring B (C1/N1/C7/C6/C5/C8) displays the same chair conformation; seven-membered ring C (C5/C6/C12/C11/C10/C9/C8) adopts a screw-chair conformation; five-membered rings D (C8/C9/C13/C14/C15) and E (C10/C9/C15/C16/C17) adopt an envelope conformations. While the five-membered heterocyclic F (C1/N1/C19/C18/C2) displays an envelope conformation.

### Experimental

The title compound was isolated from the branch of *Daphniphyllum macropodum* Miq. And crystals suitable for X-ray structure analysis was obtained by slow evaporation from an acetone solution at room temperature.

### Refinement

H atoms were located geometrically with C—H distance of 0.93–0.98 Å, and refined using a riding model with *U*_iso_(H) = 1.2 *U*_eq_(C). As no significant anomalous scatterings, Friedel pairs were merged.

### Crystal data

**Table:**

C_27_H_37_NO_7_	*F*(000) = 1048
*M**_r_* = 487.58	*D*_x_ = 1.327 Mg m^−^^3^
Orthorhombic, *P*2_1_2_1_2_1_	Mo *K*α radiation, λ = 0.71073 Å
Hall symbol: P 2ac 2ab	Cell parameters from 4404 reflections
*a* = 9.5980 (3) Å	θ = 3.0–27.0°
*b* = 9.7437 (2) Å	µ = 0.10 mm^−^^1^
*c* = 26.0986 (6) Å	*T* = 294 K
*V* = 2440.74 (11) Å^3^	Block, colorless
*Z* = 4	0.60 × 0.45 × 0.20 mm

### Data collection

**Table:**

Oxford Xcalibur diffractometer with an Eos CCD detector	2236 reflections with *I* > 2σ(*I*)
Radiation source: fine-focus sealed tube	*R*_int_ = 0.019
graphite	θ_max_ = 26.4°, θ_min_ = 3.1°
ω scans	*h* = −4→11
8335 measured reflections	*k* = −12→6
2836 independent reflections	*l* = −32→25

### Refinement

**Table:**

Refinement on *F*^2^	Primary atom site location: structure-invariant direct methods
Least-squares matrix: full	Secondary atom site location: difference Fourier map
*R*[*F*^2^ > 2σ(*F*^2^)] = 0.044	Hydrogen site location: inferred from neighbouring sites
*wR*(*F*^2^) = 0.120	H-atom parameters constrained
*S* = 1.04	*w* = 1/[σ^2^(*F*_o_^2^) + (0.0793*P*)^2^] where *P* = (*F*_o_^2^ + 2*F*_c_^2^)/3
2836 reflections	(Δ/σ)_max_ = 0.001
317 parameters	Δρ_max_ = 0.48 e Å^−^^3^
0 restraints	Δρ_min_ = −0.19 e Å^−^^3^

### Special details

**Table:**

Geometry. All e.s.d.'s (except the e.s.d. in the dihedral angle between two l.s. planes) are estimated using the full covariance matrix. The cell e.s.d.'s are taken into account individually in the estimation of e.s.d.'s in distances, angles and torsion angles; correlations between e.s.d.'s in cell parameters are only used when they are defined by crystal symmetry. An approximate (isotropic) treatment of cell e.s.d.'s is used for estimating e.s.d.'s involving l.s. planes.
Refinement. Refinement of *F*^2^ against ALL reflections. The weighted *R*-factor *wR* and goodness of fit *S* are based on *F*^2^, conventional *R*-factors *R* are based on *F*, with *F* set to zero for negative *F*^2^. The threshold expression of *F*^2^ > σ(*F*^2^) is used only for calculating *R*-factors(gt) *etc*. and is not relevant to the choice of reflections for refinement. *R*-factors based on *F*^2^ are statistically about twice as large as those based on *F*, and *R*- factors based on ALL data will be even larger.

### Fractional atomic coordinates and isotropic or equivalent isotropic displacement parameters (Å^2^)

**Table:**

	*x*	*y*	*z*	*U*_iso_*/*U*_eq_	
O1	0.6702 (2)	0.3147 (2)	0.46602 (7)	0.0382 (5)	
H1	0.7380	0.3170	0.4469	0.057*	
O2	0.1503 (2)	0.46110 (19)	0.39329 (7)	0.0362 (5)	
O3	−0.0227 (3)	0.3209 (3)	0.41677 (10)	0.0737 (8)	
O4	0.1867 (2)	0.2863 (2)	0.31031 (7)	0.0404 (5)	
O5	0.1503 (4)	0.0913 (3)	0.26965 (11)	0.0921 (11)	
O6	0.3018 (3)	−0.1150 (3)	0.40070 (9)	0.0687 (7)	
O7	0.4426 (3)	−0.0900 (3)	0.33499 (9)	0.0768 (9)	
N1	0.6017 (2)	0.4877 (2)	0.41036 (8)	0.0332 (5)	
C1	0.5508 (3)	0.3699 (3)	0.43982 (9)	0.0295 (6)	
C2	0.4577 (3)	0.4292 (3)	0.48146 (9)	0.0342 (6)	
H2	0.4451	0.3630	0.5094	0.041*	
C3	0.3173 (3)	0.4728 (3)	0.45943 (10)	0.0380 (7)	
H3B	0.2505	0.4779	0.4873	0.046*	
H3A	0.3269	0.5645	0.4453	0.046*	
C4	0.2579 (3)	0.3794 (3)	0.41792 (9)	0.0314 (6)	
H4	0.2134	0.3006	0.4346	0.038*	
C5	0.3605 (3)	0.3247 (2)	0.37656 (9)	0.0273 (5)	
C6	0.4181 (3)	0.4432 (3)	0.34171 (10)	0.0327 (6)	
H6	0.3356	0.4956	0.3313	0.039*	
C12	0.4869 (3)	0.3971 (3)	0.29090 (9)	0.0395 (7)	
H12B	0.4272	0.3283	0.2754	0.047*	
H12A	0.4882	0.4755	0.2680	0.047*	
C11	0.6344 (3)	0.3386 (4)	0.29319 (10)	0.0462 (8)	
H11B	0.6622	0.3129	0.2588	0.055*	
H11A	0.6969	0.4107	0.3045	0.055*	
C10	0.6540 (3)	0.2175 (3)	0.32725 (10)	0.0381 (7)	
C17	0.7564 (4)	0.1055 (4)	0.31502 (12)	0.0595 (10)	
H17A	0.8510	0.1392	0.3185	0.071*	
H17B	0.7433	0.0737	0.2801	0.071*	
C16	0.7302 (4)	−0.0094 (4)	0.35225 (12)	0.0604 (10)	
H16A	0.8171	−0.0456	0.3655	0.073*	
H16B	0.6784	−0.0832	0.3361	0.073*	
C15	0.6441 (3)	0.0580 (3)	0.39524 (11)	0.0411 (7)	
H15	0.7077	0.0796	0.4235	0.049*	
C14	0.5100 (3)	−0.0001 (3)	0.41841 (11)	0.0389 (7)	
H14	0.5328	−0.0641	0.4461	0.047*	
C13	0.4398 (3)	0.1315 (3)	0.44104 (9)	0.0336 (6)	
H13A	0.4709	0.1465	0.4760	0.040*	
H13B	0.3393	0.1213	0.4412	0.040*	
C8	0.4837 (3)	0.2571 (2)	0.40589 (9)	0.0268 (6)	
C9	0.5948 (3)	0.1916 (3)	0.37249 (10)	0.0316 (6)	
C19	0.6329 (4)	0.5914 (3)	0.45071 (11)	0.0467 (8)	
H19B	0.6076	0.6824	0.4388	0.056*	
H19A	0.7316	0.5910	0.4586	0.056*	
C18	0.5466 (3)	0.5536 (3)	0.49960 (11)	0.0438 (7)	
H18	0.6119	0.5204	0.5256	0.053*	
C7	0.5085 (3)	0.5450 (3)	0.37085 (11)	0.0406 (7)	
H7B	0.5651	0.5943	0.3462	0.049*	
H7A	0.4477	0.6114	0.3872	0.049*	
C20	0.4694 (5)	0.6767 (4)	0.52205 (14)	0.0678 (11)	
H20A	0.4126	0.6471	0.5502	0.102*	
H20C	0.4114	0.7171	0.4962	0.102*	
H20B	0.5357	0.7434	0.5339	0.102*	
C21	0.0177 (3)	0.4231 (3)	0.39505 (10)	0.0393 (7)	
C22	−0.0720 (3)	0.5211 (4)	0.36612 (12)	0.0503 (8)	
H22B	−0.1680	0.5048	0.3744	0.076*	
H22C	−0.0581	0.5082	0.3300	0.076*	
H22A	−0.0476	0.6134	0.3753	0.076*	
C23	0.2849 (3)	0.2182 (3)	0.34440 (10)	0.0339 (6)	
H23B	0.3518	0.1661	0.3244	0.041*	
H23A	0.2354	0.1549	0.3666	0.041*	
C24	0.1298 (3)	0.2110 (3)	0.27347 (10)	0.0407 (7)	
C25	0.0380 (4)	0.2931 (4)	0.24032 (11)	0.0509 (8)	
H25C	−0.0115	0.2334	0.2174	0.076*	
H25B	0.0930	0.3567	0.2208	0.076*	
H25A	−0.0273	0.3427	0.2611	0.076*	
C26	0.4157 (4)	−0.0727 (3)	0.37809 (12)	0.0462 (8)	
C27	0.2020 (5)	−0.1746 (4)	0.36432 (14)	0.0790 (12)	
H27B	0.2100	−0.2728	0.3648	0.118*	
H27C	0.2217	−0.1413	0.3305	0.118*	
H27A	0.1091	−0.1487	0.3739	0.118*	

### Atomic displacement parameters (Å^2^)

**Table:**

	*U*^11^	*U*^22^	*U*^33^	*U*^12^	*U*^13^	*U*^23^
O1	0.0288 (10)	0.0501 (11)	0.0357 (9)	−0.0001 (11)	−0.0087 (8)	0.0011 (9)
O2	0.0214 (9)	0.0407 (10)	0.0465 (11)	0.0010 (9)	−0.0012 (8)	0.0061 (9)
O3	0.0356 (13)	0.0947 (19)	0.0909 (18)	−0.0145 (15)	−0.0098 (13)	0.0483 (17)
O4	0.0387 (11)	0.0407 (10)	0.0417 (10)	0.0026 (11)	−0.0183 (9)	−0.0028 (9)
O5	0.124 (3)	0.0572 (15)	0.0951 (19)	0.020 (2)	−0.068 (2)	−0.0273 (15)
O6	0.0680 (17)	0.0769 (17)	0.0612 (13)	−0.0202 (16)	0.0013 (13)	−0.0102 (13)
O7	0.087 (2)	0.097 (2)	0.0469 (13)	−0.0159 (19)	0.0028 (14)	−0.0190 (14)
N1	0.0290 (12)	0.0368 (12)	0.0337 (11)	−0.0056 (11)	−0.0028 (10)	−0.0008 (10)
C1	0.0264 (14)	0.0326 (13)	0.0295 (12)	−0.0026 (12)	−0.0045 (10)	0.0003 (11)
C2	0.0357 (15)	0.0407 (15)	0.0263 (12)	−0.0004 (15)	−0.0025 (11)	−0.0016 (11)
C3	0.0315 (14)	0.0489 (16)	0.0337 (13)	0.0044 (14)	0.0017 (12)	−0.0054 (13)
C4	0.0242 (13)	0.0365 (14)	0.0334 (13)	−0.0002 (12)	0.0003 (11)	0.0068 (12)
C5	0.0250 (13)	0.0295 (12)	0.0273 (11)	−0.0013 (12)	−0.0020 (10)	0.0023 (11)
C6	0.0270 (14)	0.0394 (14)	0.0318 (13)	0.0008 (14)	−0.0029 (11)	0.0093 (12)
C12	0.0400 (16)	0.0497 (16)	0.0288 (13)	−0.0013 (16)	−0.0002 (12)	0.0097 (13)
C11	0.0383 (17)	0.067 (2)	0.0333 (14)	0.0026 (18)	0.0104 (13)	0.0074 (14)
C10	0.0314 (15)	0.0524 (16)	0.0305 (12)	0.0035 (16)	0.0028 (12)	−0.0027 (13)
C17	0.054 (2)	0.078 (2)	0.0459 (16)	0.025 (2)	0.0068 (16)	−0.0050 (17)
C16	0.057 (2)	0.064 (2)	0.0599 (19)	0.024 (2)	0.0079 (18)	−0.0019 (18)
C15	0.0401 (17)	0.0439 (15)	0.0392 (14)	0.0125 (16)	−0.0056 (13)	0.0039 (13)
C14	0.0404 (17)	0.0360 (14)	0.0404 (15)	0.0080 (15)	−0.0078 (13)	0.0043 (13)
C13	0.0374 (16)	0.0346 (13)	0.0287 (12)	−0.0016 (13)	−0.0036 (11)	0.0043 (12)
C8	0.0260 (13)	0.0311 (12)	0.0235 (12)	−0.0009 (12)	−0.0037 (10)	0.0013 (10)
C9	0.0275 (13)	0.0361 (13)	0.0313 (13)	0.0042 (13)	−0.0051 (11)	−0.0010 (12)
C19	0.0461 (19)	0.0435 (15)	0.0506 (16)	−0.0104 (16)	−0.0060 (15)	−0.0084 (14)
C18	0.0455 (18)	0.0479 (17)	0.0381 (14)	−0.0005 (16)	−0.0063 (14)	−0.0115 (14)
C7	0.0438 (17)	0.0350 (14)	0.0429 (15)	−0.0026 (15)	−0.0021 (14)	0.0063 (13)
C20	0.072 (3)	0.062 (2)	0.069 (2)	−0.002 (2)	0.001 (2)	−0.0308 (19)
C21	0.0271 (15)	0.0557 (17)	0.0352 (14)	−0.0062 (15)	0.0004 (12)	0.0056 (14)
C22	0.0322 (16)	0.066 (2)	0.0526 (18)	0.0039 (17)	−0.0034 (14)	0.0042 (17)
C23	0.0349 (15)	0.0344 (13)	0.0326 (12)	0.0024 (14)	−0.0091 (12)	0.0024 (12)
C24	0.0372 (16)	0.0472 (16)	0.0377 (14)	−0.0017 (16)	−0.0084 (13)	−0.0042 (14)
C25	0.052 (2)	0.0589 (18)	0.0421 (16)	−0.0022 (19)	−0.0180 (15)	0.0047 (15)
C26	0.060 (2)	0.0328 (14)	0.0455 (18)	0.0046 (16)	0.0151 (16)	0.0020 (14)
C27	0.073 (3)	0.075 (3)	0.089 (3)	−0.017 (2)	−0.021 (2)	−0.022 (2)

### Geometric parameters (Å, °)

**Table:**

O1—C1	1.439 (3)	C17—H17A	0.9700
O1—H1	0.8200	C17—H17B	0.9700
O2—C21	1.327 (4)	C16—C15	1.540 (4)
O2—C4	1.454 (3)	C16—H16A	0.9700
O3—C21	1.209 (4)	C16—H16B	0.9700
O4—C24	1.327 (3)	C15—C9	1.508 (4)
O4—C23	1.456 (3)	C15—C14	1.531 (5)
O5—C24	1.187 (4)	C15—H15	0.9800
O6—C26	1.309 (4)	C14—C26	1.558 (5)
O6—C27	1.468 (4)	C14—C13	1.564 (4)
O7—C26	1.166 (4)	C14—H14	0.9800
N1—C1	1.465 (4)	C13—C8	1.586 (4)
N1—C7	1.475 (4)	C13—H13A	0.9700
N1—C19	1.490 (4)	C13—H13B	0.9700
C1—C2	1.521 (4)	C8—C9	1.518 (4)
C1—C8	1.552 (4)	C19—C18	1.565 (4)
C2—C3	1.526 (4)	C19—H19B	0.9700
C2—C18	1.556 (4)	C19—H19A	0.9700
C2—H2	0.9800	C18—C20	1.527 (4)
C3—C4	1.526 (4)	C18—H18	0.9800
C3—H3B	0.9700	C7—H7B	0.9700
C3—H3A	0.9700	C7—H7A	0.9700
C4—C5	1.555 (4)	C20—H20A	0.9600
C4—H4	0.9800	C20—H20C	0.9600
C5—C23	1.520 (4)	C20—H20B	0.9600
C5—C8	1.555 (3)	C21—C22	1.491 (4)
C5—C6	1.570 (3)	C22—H22B	0.9600
C6—C7	1.522 (4)	C22—H22C	0.9600
C6—C12	1.548 (4)	C22—H22A	0.9600
C6—H6	0.9800	C23—H23B	0.9700
C12—C11	1.528 (4)	C23—H23A	0.9700
C12—H12B	0.9700	C24—C25	1.471 (4)
C12—H12A	0.9700	C25—H25C	0.9600
C11—C10	1.489 (4)	C25—H25B	0.9600
C11—H11B	0.9700	C25—H25A	0.9600
C11—H11A	0.9700	C27—H27B	0.9600
C10—C9	1.335 (4)	C27—H27C	0.9600
C10—C17	1.502 (4)	C27—H27A	0.9600
C17—C16	1.504 (5)		
			
C1—O1—H1	109.5	C15—C14—C13	102.0 (2)
C21—O2—C4	120.9 (2)	C26—C14—C13	112.1 (2)
C24—O4—C23	117.2 (2)	C15—C14—H14	109.8
C26—O6—C27	112.2 (3)	C26—C14—H14	109.8
C1—N1—C7	117.4 (2)	C13—C14—H14	109.8
C1—N1—C19	103.1 (2)	C14—C13—C8	107.5 (2)
C7—N1—C19	111.0 (2)	C14—C13—H13A	110.2
O1—C1—N1	106.1 (2)	C8—C13—H13A	110.2
O1—C1—C2	105.67 (19)	C14—C13—H13B	110.2
N1—C1—C2	105.9 (2)	C8—C13—H13B	110.2
O1—C1—C8	109.7 (2)	H13A—C13—H13B	108.5
N1—C1—C8	113.19 (19)	C9—C8—C1	109.5 (2)
C2—C1—C8	115.6 (2)	C9—C8—C5	115.43 (18)
C1—C2—C3	110.8 (2)	C1—C8—C5	107.3 (2)
C1—C2—C18	101.0 (2)	C9—C8—C13	101.2 (2)
C3—C2—C18	112.4 (2)	C1—C8—C13	109.08 (18)
C1—C2—H2	110.7	C5—C8—C13	114.2 (2)
C3—C2—H2	110.7	C10—C9—C15	112.2 (3)
C18—C2—H2	110.7	C10—C9—C8	136.7 (3)
C4—C3—C2	115.5 (2)	C15—C9—C8	111.0 (2)
C4—C3—H3B	108.4	N1—C19—C18	108.1 (2)
C2—C3—H3B	108.4	N1—C19—H19B	110.1
C4—C3—H3A	108.4	C18—C19—H19B	110.1
C2—C3—H3A	108.4	N1—C19—H19A	110.1
H3B—C3—H3A	107.5	C18—C19—H19A	110.1
O2—C4—C3	104.6 (2)	H19B—C19—H19A	108.4
O2—C4—C5	109.29 (19)	C20—C18—C2	117.6 (3)
C3—C4—C5	117.4 (2)	C20—C18—C19	112.6 (3)
O2—C4—H4	108.4	C2—C18—C19	103.0 (2)
C3—C4—H4	108.4	C20—C18—H18	107.7
C5—C4—H4	108.4	C2—C18—H18	107.7
C23—C5—C8	110.21 (19)	C19—C18—H18	107.7
C23—C5—C4	108.4 (2)	N1—C7—C6	116.7 (2)
C8—C5—C4	106.53 (18)	N1—C7—H7B	108.1
C23—C5—C6	110.51 (19)	C6—C7—H7B	108.1
C8—C5—C6	109.2 (2)	N1—C7—H7A	108.1
C4—C5—C6	111.9 (2)	C6—C7—H7A	108.1
C7—C6—C12	112.0 (2)	H7B—C7—H7A	107.3
C7—C6—C5	113.0 (2)	C18—C20—H20A	109.5
C12—C6—C5	115.7 (2)	C18—C20—H20C	109.5
C7—C6—H6	105.0	H20A—C20—H20C	109.5
C12—C6—H6	105.0	C18—C20—H20B	109.5
C5—C6—H6	105.0	H20A—C20—H20B	109.5
C11—C12—C6	118.0 (2)	H20C—C20—H20B	109.5
C11—C12—H12B	107.8	O3—C21—O2	123.6 (3)
C6—C12—H12B	107.8	O3—C21—C22	125.4 (3)
C11—C12—H12A	107.8	O2—C21—C22	110.9 (3)
C6—C12—H12A	107.8	C21—C22—H22B	109.5
H12B—C12—H12A	107.1	C21—C22—H22C	109.5
C10—C11—C12	115.8 (2)	H22B—C22—H22C	109.5
C10—C11—H11B	108.3	C21—C22—H22A	109.5
C12—C11—H11B	108.3	H22B—C22—H22A	109.5
C10—C11—H11A	108.3	H22C—C22—H22A	109.5
C12—C11—H11A	108.3	O4—C23—C5	109.6 (2)
H11B—C11—H11A	107.4	O4—C23—H23B	109.8
C9—C10—C11	128.6 (3)	C5—C23—H23B	109.8
C9—C10—C17	109.2 (3)	O4—C23—H23A	109.8
C11—C10—C17	122.1 (3)	C5—C23—H23A	109.8
C10—C17—C16	107.1 (3)	H23B—C23—H23A	108.2
C10—C17—H17A	110.3	O5—C24—O4	122.4 (3)
C16—C17—H17A	110.3	O5—C24—C25	125.8 (3)
C10—C17—H17B	110.3	O4—C24—C25	111.8 (3)
C16—C17—H17B	110.3	C24—C25—H25C	109.5
H17A—C17—H17B	108.5	C24—C25—H25B	109.5
C17—C16—C15	104.1 (3)	H25C—C25—H25B	109.5
C17—C16—H16A	110.9	C24—C25—H25A	109.5
C15—C16—H16A	110.9	H25C—C25—H25A	109.5
C17—C16—H16B	110.9	H25B—C25—H25A	109.5
C15—C16—H16B	110.9	O7—C26—O6	125.0 (4)
H16A—C16—H16B	109.0	O7—C26—C14	126.0 (3)
C9—C15—C14	102.1 (2)	O6—C26—C14	108.9 (2)
C9—C15—C16	104.5 (2)	O6—C27—H27B	109.5
C14—C15—C16	125.6 (3)	O6—C27—H27C	109.5
C9—C15—H15	107.8	H27B—C27—H27C	109.5
C14—C15—H15	107.8	O6—C27—H27A	109.5
C16—C15—H15	107.8	H27B—C27—H27A	109.5
C15—C14—C26	112.9 (2)	H27C—C27—H27A	109.5

### Hydrogen-bond geometry (Å, °)

**Table:**

*D*—H···*A*	*D*—H	H···*A*	*D*···*A*	*D*—H···*A*
O1—H1···O3^i^	0.82	2.43	3.216 (3)	161
C22—H22B···N1^ii^	0.96	2.41	3.354 (4)	169

Symmetry codes: (i) *x*+1, *y*, *z*; (ii) *x*−1, *y*, *z*.
